# Estrogen and progesterone play pivotal roles in endothelial progenitor cell proliferation

**DOI:** 10.1186/1477-7827-10-2

**Published:** 2012-01-17

**Authors:** Yuko Matsubara, Keiichi Matsubara

**Affiliations:** 1 Department of Obstetrics and Gynecology, Ehime Prefectural Niihama Hospital, Hongo, Niihama, Ehime, 792-0042 Japan

**Keywords:** menstrual cycle, neovascularization, ovarian hormones

## Abstract

**Background:**

It has been previously suggested that angiogenesis occurs during the menstrual cycle. Moreover, a rise in uterine blood flow is largely maintained by vasodilatation and substantial increases in angiogenesis. It is known that estradiol (E2) and progesterone (P4) are involved in angiogenesis. Recently, endothelial progenitor cells (EPCs) were found to be involved in neovascularization; however, their roles in uterine neovascularization have not been well characterized. We hypothesized that E2- or P4-mediated EPC proliferation plays important roles in uterine neovascularization during the menstrual cycle.

**Methods:**

The number of EPCs in peripheral blood from subjects in the menstrual phase (n = 12), follicular phase (n = 8), and luteal phase (n = 16), was measured using flow cytometry. Peripheral blood mononuclear cells (PBMCs) were cultured for seven days with or without 17beta-estradiol (E2beta) or P4, followed by assessment of EPC proliferation based upon the uptake of acetylated low density lipoprotein (LDL) and lectin. The expression of estrogen receptor (ER) or progesterone receptor (PR) in EPCs was also evaluated using real-time PCR.

**Results:**

E2beta and P4 significantly increased the proliferation of EPCs derived from the peripheral blood of subjects in menstrual phase, but not subjects in the luteal phase. In addition, the expression level of ERalpha was markedly higher than ERbeta in EPCs derived from women in menstrual phase.

**Conclusions:**

EPC proliferation is induced during the menstrual phase and proliferation can be affected by estrogen through ERalpha activation.

## Background

Angiogenesis in female reproductive organs, including the uterus, corpus luteum, and placenta, is essential for implantation and is critical for the dramatic (30-50 fold) elevation of uterine blood flow during pregnancy [1,2]. Disturbances in uterine vascular development are associated with pregnancy loss, preeclampsia, and intrauterine growth restriction [3]. Periodic uterine endometrial neovascularization begins after menstruation and continues into the luteal phase [4]. In general, it is thought that neovascularization is mainly caused by angiogenesis, which is the sprouting of capillaries from pre-existing vessels, such as in tumors and embryos. However, vasculogenesis, which is mediated by endothelial progenitor cells (EPCs), has recently been proposed to be involved in endometrial neovascularization [5,6]. The presence of EPCs in peripheral blood provides a maintenance reservoir of endothelial cells (ECs) and contributes to up to 25% of ECs in newly formed vessels [7]. It has been hypothesized that EPCs may be involved in the growth of the uterine endometrium since EPCs localize within the vasculature and stroma of the uterine endometrium and myometrium after ovulation [8].

In female reproductive organs, neovascularization may be partially regulated by cyclic changes in sex steroids, such as estradiol (E_2_) and progesterone (P_4_) [9,10] in response to the hormones produced by the hypothalamus, pituitary gland, and ovaries [11]. Serum E_2 _and P_4 _concentrations are very low during the early follicular phase [12]. During this phase, serum estrogen levels rise in parallel to the growth of follicle size and granulosa cells. Before ovulation, follicle-produced E_2 _is increased and luteinization of the granulosa cells is stimulated, which leads to the synthesis of P_4_. After ovulation, the granulosa cells continue to enlarge and become lutein. The corpus luteum, which consists of luteinized granulosa cells and theca-lutein cells, secretes P_4_. There is a secondary rise in E_2 _levels during the mid-luteal phase following a decrease before menstruation, and this rise in E_2 _levels occurs in parallel with the rise of serum P_4 _levels. However, the effects of these hormones and their interactions in reproductive organs remain unclear, especially in vasculogenesis.

Estrogen receptors (ERs) are expressed in uterine arterial ECs [13,14], as well as in other types of ECs [15], which suggests that 17β-estradiol (E_2_β) can act directly on the cells and alter uterine vascular function. It has been reported that E_2_β can enhance angiogenesis [16,17] and vasculogenesis by increasing the number of EPCs [18]. These data support the theory that E_2_β is an important factor in promoting neovascularization in female reproductive organs. Similar to E_2_β, P_4 _has also been shown to influence uterine angiogenesis [19]. P_4 _may enhance E_2_β-induced angiogenesis by increasing endothelial nitric oxide synthase (eNOS) expression [20]. In contrast, it has been reported that P_4 _inhibits E_2_β-reduced neointimal proliferation [21] and decreases ER and P_4 _receptor (PR) expression in human uterine vascular endothelium [22], which consequently attenuates E_2_β-induced angiogenic responses [23,24].

The aim of this study was to evaluate the influence of sex steroids on the proliferation of EPCs during the menstrual cycle. The findings of this study could help elucidate the role of vasculogenesis in cyclic endometrial neovascularization.

## Methods

### EPC isolation

This study was approved by the Ehime University Institutional Review Board. All participants gave informed consent for participation in this study. Peripheral venous blood (20 ml) from healthy young volunteers in the menstrual phase (n = 12; 30 ± 4 years-old), follicular phase (n = 8; 30 ± 5 years-old), luteal phase (n = 16; 28 ± 4 years-old) with regular menstrual cycles (24-35 d) was collected in tubes containing sodium heparin. The menstrual cycle dates were determined by measuring the basal body temperature (BBT) as reported by the patient and serum E_2 _(follicular phase > 10 pg/ml) and progesterone (follicular phase < 1.5 ng/ml; luteal phase > 5.7 ng/ml) concentrations. The follicular phase is characterized by a lower BBT [11]. Peripheral blood mononuclear cells (PBMCs) were isolated by density gradient centrifugation at 1600 rpm for 60 min with Ficoll-Paque separating solution (Amersham Pharmacia Biotech; AB, Uppsala, Sweden). The PBMC layer was collected, cells were counted, and then a fraction of the cells was resuspended in Dulbecco's phosphate-buffered saline (D-PBS: Invitrogen; Carlsbad, CA) with 1% fetal bovine serum (FBS: Invitrogen) for fluorescence-activated cell sorting (FACS) analysis. Circulating EPCs (cEPCs) were characterized by the expression of vascular endothelial growth factor receptor 2 (VEGF-R2; kinase domain receptor [KDR]), AC133, and CD34 on the surface of PBMCs.

To assess the proliferation of EPCs, the cells were cultivated according to a previously described technique [25]. Briefly, PBMCs (8 × 10^5 ^cells) were seeded into each well of 96-well culture plates coated with human fibronectin (Sigma-Aldrich, St. Louis, MO) and cultured in endothelial basal medium-2 (EBM-2, Clonetics; San Diego, CA) supplemented with EGM-2MV (Clonetics) consisting of 5% FBS, VEGF, fibroblast growth factor-2 (FGF2), epidermal growth factor (EGF), insulin-like growth factor-1 (IGF1), and ascorbic acid. After four days in culture, nonadherent cells were removed and adherent cells were subsequently cultured for an additional three days.

### Low density lipoprotein (LDL)/lectin assay

After seven days in culture, adherent cells were assessed as EPCs based on the uptake of 1,1'-dioctadecyl-3,3,3',3'-tetramethylindocarbocyanine-labeled acetylated LDL (Dil-Ac-LDL, Biogenesis; Poole, UK) and FITC-labeled *Ulex europaeus *agglutinin I (FITC-lectin, Sigma-Aldrich). Cells were incubated with Dil-Ac-LDL (2.4 μg/ml) for one hour at 37°C and fixed with 2% paraformaldehyde for 10 min. Cells were washed twice with DPBS and then incubated with FITC-lectin (10 μg/ml) for one hour at 37°C. Cells were washed twice with DPBS and detached with 0.25% trypsin/EDTA.

### FACS analysis

**Figure 1:**
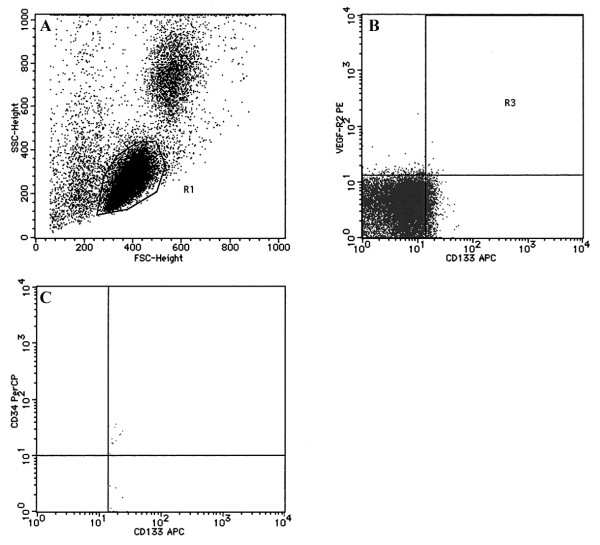
**Characterization of circulating EPCs from peripheral blood**. A) The mononuclear cell fraction was selected based on low granularity by forward and side scatter using FACS. B, C) Sorted cells were fixed and immunostained with antibodies against human KDR, AC133, and CD34, and then subjected to FACS analysis.

**Figure 2:**
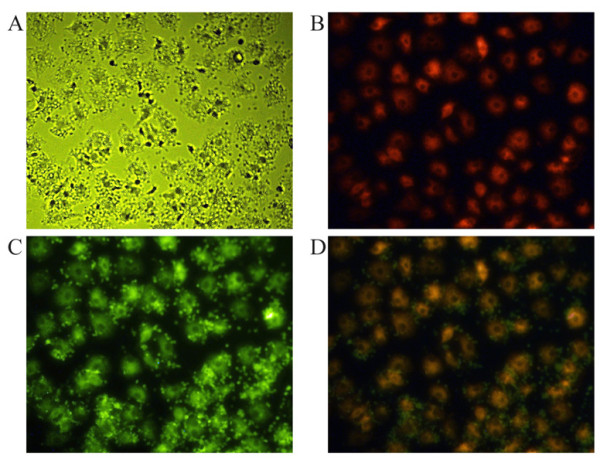
**LDL/lectin assay to detect EPCs in culture**. A) Cultivated PBMCs were examined using Dil-Ac-LDL/FITC-lectin uptake assays. B) Live EPCs exposed to Dil-AcLDL and C) stained with FITC-lectin (*Ulex europeaus*) D) Merged images (40 × final magnification).

Before culturing the cells, the cEPCs were confirmed by FACS with a PE-labeled monoclonal antibody against human KDR (Genzyme Techne; Cambridge, MA), a FITC-labeled monoclonal antibody against human AC133 (Genzyme Techne), and a PerCP-labeled monoclonal antibody against human CD34 (Becton Dickinson). Then, 1.0 × 10^5 ^PBMCs were incubated with the three antibodies for 30 min at 4°C in the dark. Isotype antibodies were used as controls. After incubation, cells were analyzed with a FACScan flow cytometer (Becton Dickinson) and Cell Quest software (Becton Dickinson) (Figure 1). On the 7th day of culture, adherent cells were incubated with Dil-acLDL and FITC-lectin, and double-stained cells were analyzed by FACS (Figure 2).

### EPC culture with E_2_β or P_4_

To assess the effects of E_2_β or P_4 _on EPC proliferation of EPCs, 8 × 10^5 ^PBMCs, derived from the peripheral blood of women in the menstrual or luteal phases, were seeded into each well of 96-well culture plates coated with human fibronectin (Sigma-Aldrich), and cultured in endothelial basal medium (phenol red free EBM-2, Clonetics; San Diego, CA) supplemented with EGM-2MV (Clonetics) consisting of 5% charcoal stripped serum (Invitrogen), VEGF, FGF2, EGF, IGF1, and ascorbic acid with or without 10^-9^-10^-7 ^M of E_2_β or P_4_. ICI 182,780 (10^-5 ^M, Wako Pure Chemical Industries, Ltd., Osaka, Japan) or RU486 (10^-5 ^M, Sigma-Aldrich) was used as an inhibitor of ER or PR, respectively [26]. After four days in culture, nonadherent cells were removed and adherent cells were subsequently cultured for an additional three days.

### Isolation of RNA and semi-quantitative RT-PCR

Total RNA was collected from cells grown in 3.5 cm cell culture dishes. Cells were washed with cold DPBS and subsequently frozen at -80°C until isolation of total RNA. The cells were disrupted and homogenized with an ultrasound homogenizer. Total RNA was extracted using the RNeasy Mini Kit (QIAGEN; Valencia, CA) according to the manufacturer's instructions. Total RNA (2 μg) was reverse transcribed to cDNA by incubating the samples with a random primer (100 pmol/μl) at 70°C for 10 min, followed by 4°C for 10 min. The samples were then incubated with Tris-HCl (pH 8.3), 75 mM KCl, 3 mM MgCl_2_, 10 mM DTT, 2.5 mM dNTPs, ribonuclease inhibitor (40 U/λ Promega Corp. Madison, WI), AMV reverse transcriptase XL (37 U/λ Takara Biochemicals Tokyo, Japan), and a dNTP mixture (2.5 mM) and then incubated at 42°C for one hour. The samples were subsequently heated at 99°C for 5 min to terminate the reaction, and stored at 4°C.

ERα, ERβ, progesterone receptor AB (PR AB), progesterone receptor B (PR B), and glyceraldehyde-3-phosphate dehydrogenase (GAPDH) oligonucleotide primers were constructed from published nucleotide sequence databases (Table 1). The level of GAPDH mRNA served as an internal standard for normalization of ER and PR mRNA levels. RT-PCR conditions were optimized to ensure that amplification proceeded within the linear portion of the reaction. The semi-quantitative RT-PCR amplification profile consisted of denaturation at 95°C for 3 min followed by 35 cycles of 95°C for 10 s and 65°C for 1 min.

**Table 1:** List of oligonucleotide primers used.

**GeneBank accession No**.	mRNA	Sequence	Length of DNA product (bp)
NM000125	ERα	5' AAG AGC TGC CAG GCC TGC C 3' (sense)	168
		5' TTG GCA GCT CTC ATG TCT CC 3' (antisense)	
AB006590.1	ERβ	5' TAA AAG AAG CAT TCA AGG ACA TAA T 3' (sense)	160
		5' GCA CTT CTC TGT CTT CGT ACT ATT C 3' (antisense)	
AY382151	PR AB	5' TGG AAG AAA TGA CTG CAT CG 3' (sense)	196
		5' TAG GGC TTG GCT TTC ATT TG 3' (antisense)	
AB085683	PR B	5' ACA CCT TGC CTG AAC TTT CG 3' (sense)	196
		5' CTG TCC TTT TCT GGG GGA CT 3' (antisense)	
NM002046	GAPDH	5' CCA CCC ATG GCA AAT TCC ATG GCA 3' (sense)	622
		5' TCT AGA CGG CAG GTC AGG TCC ACC 3' (antisense)	

The iQ SYBR Green Supermix (QIAGEN), 0.2 μM of sense and antisense oligonucleotides (ER, PR, or GAPDH), and 0.1 μg cDNA were used in a final volume of 30 μl for each RT-PCR reaction. The PCR products were digitally analyzed using a luminescent image analyzer (FluoImager, Beckton Dickinson) and quantified using Image Quant software (Beckton Dickinson).

### Statistical analysis

The results were expressed as mean ± standard error (SE). Statistical analyses were performed by one-way analysis of variance (ANOVA) followed by a *post-hoc *test (Bonferroni). Statistical significance was determined as *p *< 0.05.

## Results

### Detection of cEPCs in peripheral blood during the menstrual cycle

**Figure 3:**
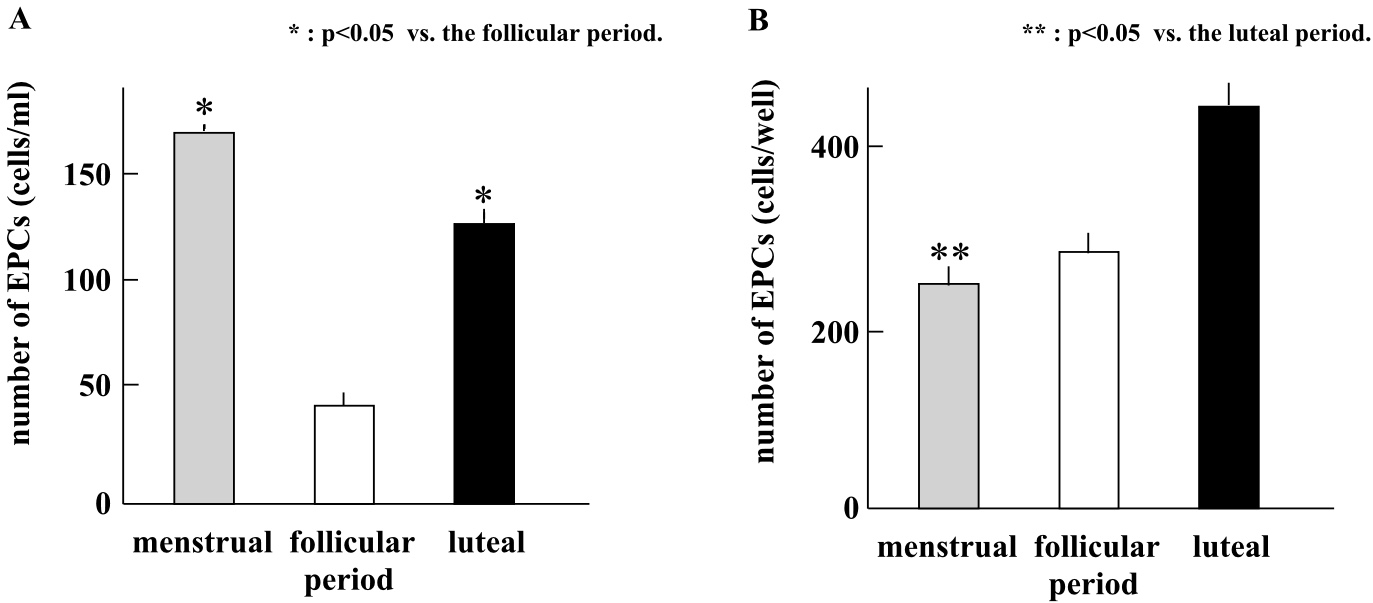
**Changes in circulatory EPC proliferation during the menstrual cycle**. A) The number of EPCs in peripheral blood. *: *p *< 0.05 vs. the follicular phase. B) The proliferation of EPCs on the 7th day in culture. **: *p *< 0.05 vs. the luteal phase.

The number of cEPCs in the peripheral blood obtained from subjects in the menstrual cycle was significantly increased during the luteal (124.0 ± 29.1 cells/ml) and menstrual (165.3 ± 38.5 cells/ml) phases compared to the follicular phase (44.1 ± 11.1 cells/ml; *p *< 0.05; Figure 3A).

### LDL/Lectin assay of cells cultured for seven days

The number of EPCs that were LDL^+^/lectin^+ ^derived from the peripheral blood of subjects in the luteal phase was significantly increased on the seventh day of culture (481.2 ± 91.2 cells/well) compared to the menstrual phase (227.1 ± 26.5 cells/well; *p *< 0.05; Figure 3B).

### Effects of E_2_β and P_4 _on the proliferation of EPCs in peripheral blood

**Figure 4:**
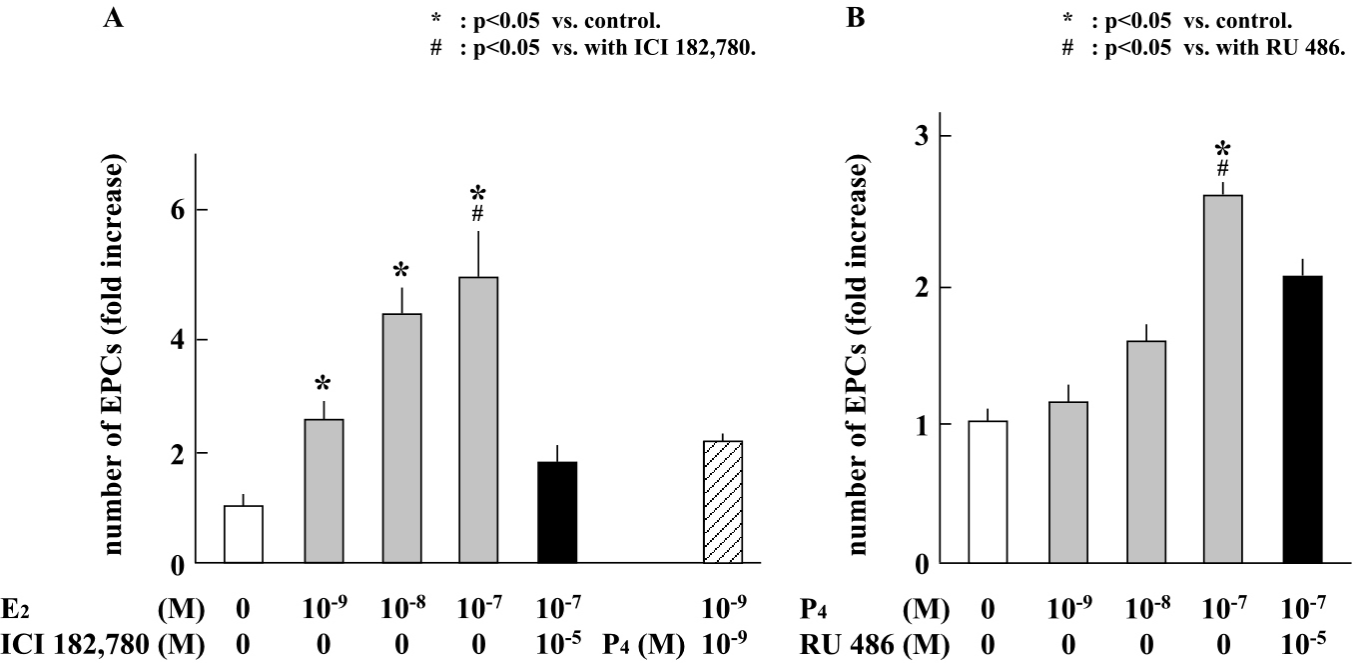
**Effects of E_2_β or P_4 _on the proliferation of EPCs from menstrual phase**. A) The effect of E_2_β on the proliferation of EPCs. B) The effect of P_4 _on the proliferation of EPCs. *: *p *< 0.05 vs. control. ^#^: *p *< 0.05 vs. cells treated with RU486.

**Figure 5:**
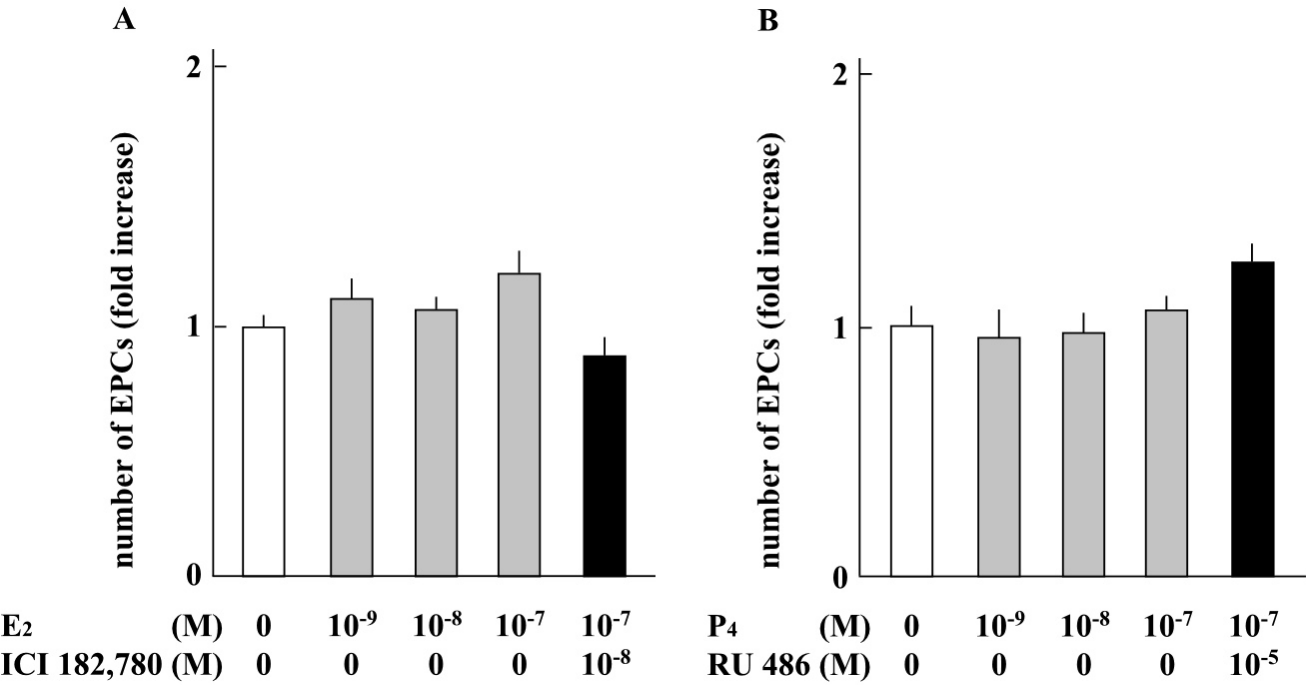
**The effect of E_2_β or P_4 _on the proliferation of EPCs from luteal phase**. A) The effect of E_2_β on the proliferation of EPCs. B) The effect of P_4 _on the EPCs proliferation.

E_2_β or P_4 _significantly increased the proliferation of EPCs derived from the peripheral blood of subjects in the menstrual phase by activating the respective receptors in a dose-dependent manner (Figure 4). Receptor antagonists of ER and PR, ICI 182,780 and RU 486, respectively, reduced the proliferation of EPCs from menstrual phase. In addition, P_4 _did not influence the effect of E_2_β on EPC proliferation. In contrast, the proliferation of EPCs from luteal phase was not influenced by E_2_β or P_4 _(Figure 5). Moreover, these receptor antagonists did not affect the proliferation of EPCs from luteal phase.

### Change in ER and PR expression in cEPCs

**Figure 6:**
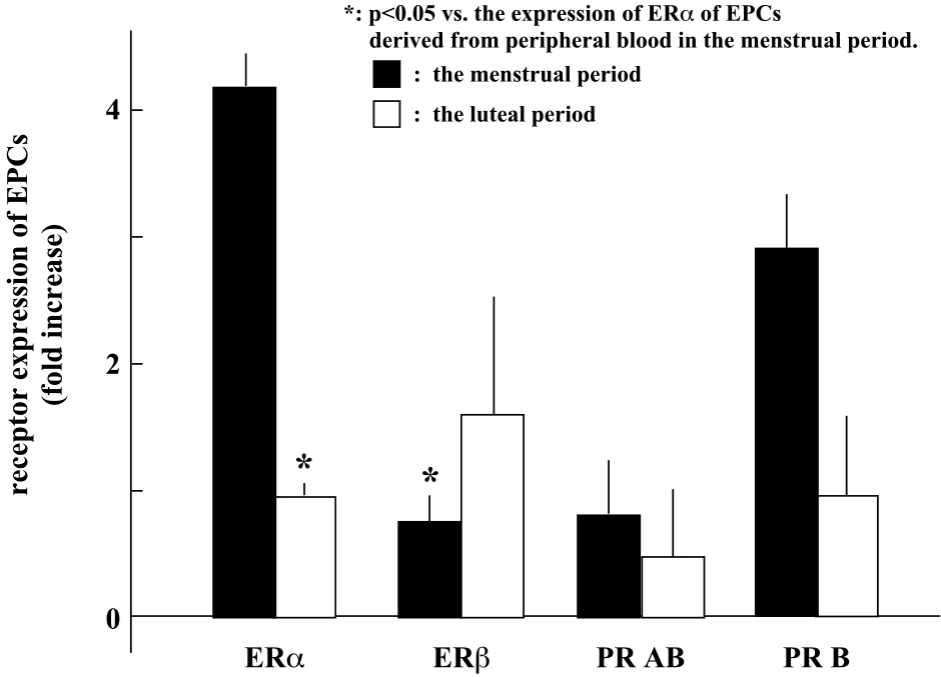
**Expression of ER and PR mRNA in circulating EPCs**. ERα, ERβ, and PR mRNA expression in EPCs derived from peripheral blood during the menstrual cycle (menstrual phase: solid column, luteal phase: open column). *: *p *< 0.05 vs. the expression of ERα in EPCs from menstrual phase.

ERα mRNA expression levels in EPCs from menstrual phase were higher than ERβ mRNA as well as ERα mRNA in EPCs from luteal phase (*p *< 0.05; Figure 6). In contrast, no significant difference was observed between the expression level of total PR mRNA and isoform B mRNA in EPCs; however, the expression levels of total PR mRNA and isoform B mRNA were limited in EPCs (Figure 6).

## Discussion

cEPCs in the peripheral blood serve as a reserve for EC maintenance, and contribute up to 25% of ECs in newly formed vessels of ischemic lesions [25,27-32]. In addition, estrogen preserves ischemic heart function during myocardial infarction by augmenting the mobilization and incorporation of bone marrow-derived EPCs into sites of neovascularization [31]. In female reproductive organs, EPCs appear to play a crucial role in vascularization of the uterine endometrium at the site of embryo implantation and placentation [33-35]. During the menstrual cycle, the number of cEPCs in peripheral blood exhibits a cyclic change, whereby it is markedly increased during the luteal and menstrual phase, and decreased during the follicular phase. The number of cEPCs has been shown to decrease over the course of pregnancy [36]. Since EPCs were also found in the corpus luteum and uterine endometrium, the estrogen-mediated increase in EPCs is thought to be involved in periodic neovascularization during the menstrual cycle [28,37,38].

The differences in EPC proliferation and cEPC concentration between the menstrual and luteal phases were the most important findings of this study. We demonstrated that EPCs from menstrual phase did not proliferate after seven days in culture; however, there was a dose-dependent increase in proliferation after cells were treated with E_2_β or P_4_. On the other hand, proliferation was increased in EPCs from luteal phase after seven days in culture; however, the proliferation was not influenced by addition of E_2_β or P_4_. Therefore, we hypothesize that luteal EPCs may have already reached maximum stimulation *in vivo *and could not be activated furtherer.

Decreased proliferation of EPCs from menstrual phase suggests that this observed effect was due to decreased concentration of E_2_β or P_4 _during menstruation. Therefore, the increase in proliferation was a sex steroid-mediated dose-dependent increase. Since the expression of ERα and ERβ in EPCs was different between the menstrual and luteal phases, E_2_β-induced neovascularization may be mediated by both receptors, and the differential expression of ERα and ERβ indicates that they have different effects on neovascularization. ERα mRNA expression levels in EPCs from menstrual phase was higher than those from luteal phase as shown in Figure 6. On the other hand, the ERβ mRNA expression level in EPCs from luteal phase was higher than ERα expression. Since estrogen-responsive element-dependent gene transcription activities are severely impaired in EPCs obtained from ERα-knockout mice, vascular growth is down-regulated in ERα-knockout EPCs [39,40], and epithelial cell proliferation can be reduced through the activation of ERβ [30], the increased expression of ERα may at least partially explain the increase in E_2_β-induced proliferation of EPCs derived from subjects in the menstrual phase. However, the roles of ERα and ERβ in EPC biology during the menstrual cycle will require further elucidation.

The cEPC concentration was lowest in the follicular phase, and EPC proliferation in the cultures was not different compared to the other cultures. It has been well established that the serum concentration of E_2 _and VEGF is very low during the early follicular phase [12,41]. Therefore, it is possible that decreased VEGF and E_2 _expression during the follicular phase could reduce the recruitment of cells from bone marrow into blood, since VEGF can induce the recruitment of EPCs from bone marrow [42]. These observations suggest that the initial priming of the ovarian hormone is required for EPC proliferation during the menstrual cycle.

Estrogen also stimulates extensive placental neovascularization through the up-regulation of angiogenic factors in the uterus during pregnancy [43], and the number of cEPCs peaks from luteal phase through the 1st trimester [44]. This finding is consistent with the fact that vasculogenesis peaks during embryogenesis [35,45]. On the other hand, estrogen is reduced after menopause, which leads to the depletion of EPCs [38], and estrogen replacement therapy can delay the onset of senescence in bone marrow-derived EPCs [46]. Therefore, estrogen is critical in promoting angiogenesis and vasculogenesis in female reproductive organs.

## Conclusion

In the female reproductive system, vasculogenesis is a recurring phenomenon controlled by the cyclical development of a transient structure and the cyclical repair of damaged tissue by E_2 _and P_4 _during the menstrual cycle. It is hypothesized that neovascularization is regulated by the crosstalk between these hormones *in utero *during the ovarian cycle and pregnancy in preparation for implantation and the maintenance of pregnancy. The findings described in this study provide evidence that the physiologic cycle of estrogen regulates EPC proliferation through the alternating balance of ER mRNA expression.

## Competing interests

The authors declare that they have no competing interests.

## Authors' contributions

YM collected all samples, carried out the molecular genetic studies, participated in the sequence alignment, and drafted the manuscript. KM carried out EPC isolation, LDL/lectin assay, and FACS analysis. KM also participated in the design of the study, performed the statistical analysis, and helped to review the manuscript. All authors read and approved the final manuscript.
